# Umbilical cord levels of macrophage migration inhibitory factor in neonatal respiratory distress syndrome

**DOI:** 10.3906/sag-2008-113

**Published:** 2021-04-30

**Authors:** Süleyman BAYRAKTAR1, Bilge TANYERİ BAYRAKTAR, Ülkan KILIÇ

**Affiliations:** 1 Department of Pediatric Intensive Care, Bezmiâlem Vakıf University, İstanbul Turkey; 2 Department of Neonatology, Bezmiâlem Vakıf University, İstanbul Turkey; 3 Department of Medical Biology, University of Health Sciences, İstanbul Turkey

**Keywords:** Macrophage migration inhibitory factor, respiratory distress syndrome, neonate, lung maturation

## Abstract

**Background/aim:**

We aimed to evaluate the association of the umbilical cord macrophage migration inhibitory factor (MIF) with the respiratory distress syndrome (RDS) in preterm infants.

**Materials and methods:**

A total of eighty six preterm infants (38 with RDS and 48 without RDS) were involved in the study. ELISA is the technique assaying MIF values.

**Results:**

The mean of the infants’ gestational ages and birth weights were significantly different (P = 0.0001). There were no significant differences in sex, delivery mode or exposure to antenatal steroid among the groups (P > 0.05). Umbilical cord MIF levels of the infants were not correlated with gestational age and birth weight (Spearman’s rho = –0.22 and 0.28 respectively, P > 0.05). There was no statistically significant difference in umbilical cord MIF levels of infants whether or not they were administered antenatal steroid (median:17.88 vs. median:17.60, Mann–Whitney U test, P = 0.42). Cord serum MIF levels were higher (mean, 17.09 ± 5.86 ng/mL) in the RDS group than in the non-RDS group (mean, 14.72 ± 4.18 ng/mL) (P = 0.005).

**Conclusion:**

This study shows that, MIF level is higher in the cord blood of the infants with RDS than of the infants without RDS. This supports that MIF expression begins in prior to the birth of the preterm infants and MIF has enhancing impact on the lung development of premature babies. With future studies, the assessment of the cord MIF levels at the bedside may be beneficial for the diagnosis and treatment of RDS, and taking actions to prevent long-term consequences.

## 1. Introduction

Respiratory distress syndrome (RDS) is the most common respiratory complication of premature infants in early life [1–5]. Main causes of RDS are underdevelopment of the lung and lack of surfactant synthesis [1–3]. Even the low birth weight (BW) and the low gestational age (GA) increase the risk of RDS, these factors alone are not enough for a prediction regarding to the development of the disease [2,3]. Identifying the molecular parameters that cause RDS and bronchopulmonary dysplasia (BPD) will lead breakthrough improvements in the treatment process [2,3,5]. 

Macrophage migration inhibitory factor (MIF) is a primary immune response regulator that has a role in the development of serious diseases, such as acute respiratory distress syndrome (ARDS), asthma, cancer and autoimmune disorders [6–12]. MIF has a devastating role in adult ARDS [10,11]. In literature search, there is only one human study about the effect of MIF on the developmental lung diseases in newborn infants [13].

It was aimed to determine the differences of MIF values in the cord blood of the preterm babies with RDS and non-RDS, and to assess whether the MIF may be a diagnostic marker for RDS in the light of future studies. 

## 2. Materials and methods

This prospective cross sectional study was conducted in a tertiary neonatal intensive care unit (NICU) of Bezmiâlem Vakıf University Hospital between July 2012 and June 2013. Since the cord blood was used, only inborn infants were included in the study. 

### 2.1. Patients

The groups were allocated into two by selecting the infants of GAs between 24 and 34 weeks. Exclusion criteria for the study were listed as: (i) infants with early onset neonatal sepsis or congenital anomalies or small for gestational age, (ii) infants who had mothers with diabetes mellitus or gestational diabetes mellitus, (iii) mothers with a history of chronic diseases such as renal failure, autoimmune diseases or hypertension, (iv) mothers who had preterm premature rupture of membranes (PPROM) longer than 48 h or had chorioamnionitis.

Group 1, included thirty-eight infants, who were diagnosed with RDS according to the clinical signs and the radiological findings. Forty-eight infants were in group 2, who had non-RDS. Six infants whose cord blood samples were not taken, two infants with Down syndrome and one infant with complex congenital heart defect were excluded from the study.

The following data were collected: BW, GA, sex, delivery mode, exposure to the steroids before the birth, the days on the respiratory support and the oxygen supplementation, and presence of BPD and the other complications of preterm birth. BPD was determined according to the guidelines [3]. 

### 2.2. Measurement of serum MIF levels

Umbilical cord blood samples (10 mL) from all premature infants were drawn into a tube under sterile conditions. The samples were taken from the umbilical cord just after the birth. It needs to be noted that the delayed cord clamping procedure was not a practice of obstetricians at the time of the study. The sample tubes were placed in ice packs and delivered to the laboratory according to the cold chain rules. Then, the umbilical cord blood was centrifuged at 4.500 rpm, and the serum was taken into Eppendorf tubes. The samples were kept in the –80 °C freezer until the working day. The samples were analysed with ELISA via MIF kit (Quantikine, R&D Systems, Inc., Minneapolis, MN, USA) (catalogue number DMF00B; R&D Systems, Inc.). MIF concentration was mentioned between 15.3 and 52.3 ng/mL by the manufacturer.

### 2.3. Statistical analysis

To analyse the data, SPSS software (version 18.0; SPSS Inc., Chicago, IL, USA) was performed. The sample distribution was tested by Kolmogorov–Smirnov considering the GA, BW and umbilical cord MIF values. Normally distributed variables were compared by t-test and expressed as the mean ± standard deviation. The categorical variables were analysed with chi-square test. The comparison of MIF values in the cord blood that were not normally distributed, was determined using the Mann–Whitney U test. The relations of GA and BW with MIF values were shown by Spearman’s correlation test. A P-value lower than 0.05 was noted statistically significant.

## 3. Results

The clinical characteristics of both groups were presented in Table 1. The mean of GA, BW, and duration of mechanical ventilation, noninvasive ventilation and oxygen use were significantly different between the infants with RDS and non-RDS (P values; 0.0001, 0.0001, 0.01, 0.004, 0.003, respectively). BW and GA were significantly lower in infants with RDS than in those without RDS. Sex, delivery mode or antenatal steroid (ANS) exposure showed no significant differences between the groups (P > 0.05). The causes for the preterm birth were detected as unknown reason (48.8%), preeclampsia (24.4%), cervical insufficiency (15.1%), PPROM (<48 h) (8.1%), placenta previa (3.6%). Antibiotics were given to mothers who experienced PPROM (n = 7). 

**Table 1:** Demographic characteristics of preterm infants.

Variable	Infants without RDS (n = 48)	Infants with RDS (n = 38)	P value
Gestational age (week)	33.40 ± 1.87	29.79 ± 3.51	0.0001
Birth weight (gram)	1981.98 ± 420.12	1353.68 ± 508.09	0.0001
Sex (Male)	22 (45.83)	23 (60.53)	NS
Antenatal steroid exposure	25 (52.10)	18 (47.40)	NS
Delivery mode (caesarean section)	32 (66.67)	31 (81.58)	NS
Apgar score, 1min, median (range)	7 (4–9)	6 (1–9)	0.004
Apgar score, 5 min, median (range)	8.5 (6–10)	8 (5–9)	0.001
Mechanical ventilation (day)	0.85 ± 5.09	8.50 ± 20.39	0.01
Noninvasive ventilation (day)	1.64 ± 3.71	6.39 ± 10.20	0.004
Oxygen use (day)	3.94 ± 9.29	20.55 ± 35.61	0.003

Cord MIF levels were higher (P = 0.005) in the RDS group (mean, 17.09 **±** 5.86 ng/mL) than in the non-RDS group (mean, 14.72 **±** 4.18 ng/mL) (Figure). MIF levels were not correlated with GA or BW (Spearman’s rho = –0.22 and 0.28 respectively; P > 0.05, Table 2). There was no statistically significant difference in umbilical cord MIF levels of infants whether or not they were administered ANS (median: 17.88 vs. median: 17.60, Mann–Whitney U test, P = 0.42). Considering to the delivery mode and cord MIF levels, we found statistically significant difference between groups of the caesarean section and the vaginal delivery (median: 18.21 and median: 11.91 respectively, Mann–Whitney U test, P = 0.000). The relationship between BPD and MIF values was not evaluated in this study. In our study, we diagnosed BPD only in eight infants. Six infants died before 36 weeks of postconceptional age so they were not evaluated for BPD. 

**Figure:**
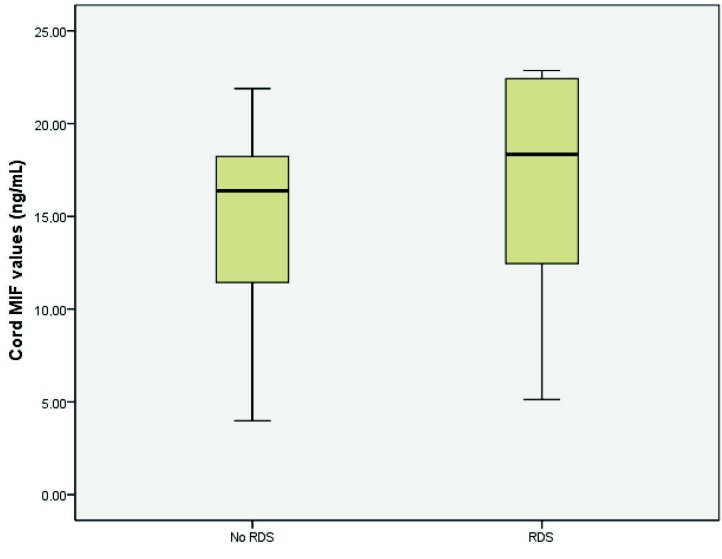
Cord blood MIF levels in preterm infants with and without respiratory distress syndrome.

**Table 2:** Correlation between gestational age, birth weight and MIF levels.

Variables (n = 86)	MIF	P
GA (r*)	–0.22	0.13**
BW (r*)	–0.28	0.06**

According to clinical follow up of our study groups, five (5.8%) babies had necrotizing enterocolitis, ten (11.6%) had patent ductus arteriosus, six (7.0%) had intraventricular haemorrhage. Of the 68 patients whose ophthalmologic screening were done, 16 (23.5%) infants had retinopathy of prematurity. 

## 4. Discussion

This study assessed that the cord MIF levels were higher in infants with RDS than in infants with non-RDS. It was emphasized that MIF could be detected in the cord blood and might be used in the early diagnosis of RDS. RDS is a multifactorial disease that involves polymorphisms of genes functioning in the lung development, the alveolar stability and the pulmonary host defence [2]. Although the major causes of RDS are undeveloped peripheral airways and lack of surfactant, the mechanisms of tissue injury have not been clarified [4]. Many factors impair respiratory adaptation and delay recovery from RDS [2]. 

MIF has a diurnal rhythm, which suggests the neuroendocrine control and it ranges between 2 and 10 ng/mL in human blood [7]. MIF has been found in serum, urine, amniotic fluid, human milk and cord blood [13–15]. It was known that MIF is secreted more rapidly in comparison to other proinflammatory mediators such as IL-1, IL-6, IL-8 and tumor necrosis factor-α (TNF-α) [15]. Therefore, the studies have focussed on the pregnancy, the labor and the infants to identify the pathogenesis of abnormal conditions [13,15]. However, the trials that measured the cord MIF levels were scarce. The cord blood MIF levels were found higher than maternal serum MIF levels in vaginal deliveries in term neonates [15]. We observed that the cord blood MIF levels were significantly higher in caesarean delivery than vaginal delivery in preterm infants. In another study, the peripheral blood level of MIF was markedly increased in the first day of life in the premature infants nevertheless they did not found any correlation between MIF levels and GA [13]. We found that there were no statistically significant differences in the MIF levels between infants with GAs 24–29 weeks and those with GAs 30–34 weeks (not shown). We also did not find any significant difference between ANS exposure and MIF levels in our study. 

MIF has pleiotropic traits that play a main role in various inflammatory diseases as well as in cancer [6–9]. Blood MIF concentrations can be elevated to extremely high levels in the inflammatory diseases [8,9]. Furthermore, MIF has been involved in the pathogenesis of sepsis, asthma, cystic fibrosis, metabolic diseases [8–10,16–19]. MIF favours the innate immune response [17]. Downregulation of MIF is a proposed therapeutic option in MIF-related disorders [16]. Calandra et al. found that MIF was in a high concentrations in septic patients [16,17]. MIF involves in embryogenesis and in parturition [20,21]. The roles and functions of MIF in neonates are not well known [22]. There are limited articles about MIF in neonates [13,23,24]. The MIF levels are increased in the acute stage of necrotising enterocolitis in premature babies [23]. 

Alveolar macrophages, bronchial epithelial cells, alveolar endothelial cells, monocytes and eosinophils expresses the MIF in lungs [11–13]. MIF seems to take a part in inflammatory lung disease [11,12]. MIF also regulated the angiogenesis [16]. The patients with ARDS had high levels of MIF in bronchoalveolar lavage [10,11]. The convincing evidence on the effective role of MIF in the pathophysiology of developmental neonatal lung diseases are limited [13,24]. MIF controlled the production of other cytokines such as IL-1β, IL-6, IL-8 and TNF-α in neonatal lung diseases [16]. It was shown that MIF played a role in lung maturation in animal studies. Prematurely born MIF-deficient mice had severe respiratory distress and mortality was high in this group [12]. It has been shown that MIF increases the expression of surfactant protein B from epithelial type II cells in the alveoli [16]. Prencipe et al. [13] found high serum MIF levels in preterm neonates with RDS on day 1 after birth. Taken together, these studies conclude that MIF is effective and protective factor in lung maturation in preterm infants [12,13]. The present study also contributes to the literature by finding high cord blood MIF levels in preterm infants with RDS just before breathing. Our outcomes support the studies that noted increased expression of MIF before and after breathing [13]. Thomas et al. [24] showed that MIF in tracheobronchial aspirate fluid was the highest in the first 24 h after birth in the extremely premature infants. Our findings also suggest that releasing of MIF is starting in the antenatal period. Whether the cord values reflect lung concentrations remains to be established.

According to the European guidelines, the early surfactant therapy is better than the late surfactant therapy in terms of the complications [1]. Today, the diagnosis of RDS can only be determined by clinical signs, using chest X-ray and failure of continuous positive airway pressure. In practice, this causes confusion for neonatologists. The efficacy of detecting surfactant in gastric aspirates is being tested [1]. Future work which will define the cut-off MIF values of patients with RDS can be useful to get a clue RDS development at bedside after birth. Thus, the surfactant can be given without clinical deterioration and the patient can be protected from the invasive interventions and the respiratory complications as well as BPD. Clinicians can also avoid the late diagnosis or giving unnecessary surfactant.

Our study also has some limitations. Firstly, we only measured the cord blood MIF so we could not compare such levels with those in peripheral blood. Secondly, a genetic investigation could not done, such as the studies of the MIF-173 SNP. Thirdly, even the MIF stimulates the expression of many proinflammatory cytokines just as IL-6, IL-8, TNF-α, the investigation on any of them could be held due to the financial issues. In fourth, the MIF values after surfactant therapy could not be studied to show the prolonged inflammatory response. In fifth, BPD was not evaluated in this study as the extremely preterm babies were not common. Lastly, the present study was not able to show that the cord levels of MIF reflect on the lung concentrations.

In conclusion, this study suggests that the MIF level is higher in the cord blood in infants suffering from RDS. This supports that MIF starts to release in intrauterine life period and MIF is an important factor to improve lung development of premature infants. With future studies, identifying the cut off values of MIF in RDS, and the assessment of the cord MIF levels at the bedside could be useful to manage the proper treatment in a timely manner and to prevent RDS and its long term consequences by taking precautions.

## Ethical approvement

The study was approved by Bezmiâlem Vakıf University’s local ethical board (B.30.2.BAV.0.05.05/385). 

## Informed consent

Informed consent was obtained from the parents.
